# Stable and destabilised states of subjective well-being: dance and movement as catalysts of transition

**DOI:** 10.1080/14649365.2014.950689

**Published:** 2015

**Authors:** Sarah Atkinson, Karen Scott

**Affiliations:** a Centre for Medical Humanities and Department of Geography, Durham University, Lower Mountjoy, Durham DH1 3LE, UK; b Centre for Rural Economy, School of Agriculture, Food and Rural Development, University of Newcastle, Newcastle NE1 7RU, UK

**Keywords:** well-being, movement, dance, health, change, destabilisation

## Abstract

The pursuit of subjective well-being has become an important object of policy and personal action, which within geography has been engaged largely by those with an interest in health. But to date, geography has given little attention to the ways in which subjective well-being changes and in particular, the ways in which it may be understood as both stable and amenable to change. Similarly, the field of arts and health asserts the value of participation in the creative arts for enhancing subjective well-being, but has also hardly addressed how this may come about. The paper explores stability and change in well-being through a case study of a dance and movement intervention in an English primary school. We draw on Deleuze and Guattari’s notions of assemblages and of striated and smooth space to explore how participation in the arts may enable escape from habituated practices. This exploration expands the scope of geographies of health towards capturing the moments and processes through which transitions in subjective well-being may occur. The study indicates the need for greater attention to gentler and gendered forms of transition.

## Setting the scene

The paper aims to question and explore the issues around how we conceptualise changes and transitions in subjective well-being and, more specifically, to reflect on how subjective well-being may, somewhat paradoxically, be comprehended as both durable and ephemeral, and stable and changeable. The paper’s argument draws on a case study of a dance and movement intervention with a primary school class that aimed to benefit both social and emotional well-being of pupils and their engagement in learning. The paper contributes specifically to the geographies of health by drawing out the different ways that subjective well-being is currently conceptualised within the sub-discipline and advocating an expanded agenda of research that opens dialogue with those geographies concerned with affective assemblage. Such dialogue promises avenues for comprehending different states of subjective well-being and particularly the transitions between them.

Over the last 20 years, well-being has become the dominant concept through which we engage with the age-old question of what constitutes a good and flourishing life. More recently, policy communities have also turned their attention to assessing subjective well-being (New Economics Foundation [NEF], 2004, 2011). In 2011, the UK government commissioned the Office for National Statistics to consult and develop measurements of subjective well-being as part of assessing social progress (Self, Thomas, & Randall, 2012), an initiative already found in a number of other countries including France, Canada, Australia and most particularly Bhutan, which has led on developing a national ‘Gross National Happiness Index’ (NEF, 2012). In an educational context, perceived childhood disadvantage in subjective social and emotional well-being has become a policy priority following evidence of an association with physical health, educational attainment (Diener & Chan, 2011; Wilkinson & Pickett, 2009) and longer-term implications for adult health (National Institute for Clinical Excellence [NICE], 2008). School-based interventions have been evaluated as a cost-effective route for health promotion and the prevention of life-long ill-health (NICE, 2008). This attention to the emotional as a priority in education is not universally welcomed; critical engagements argue that the emphasis on vulnerability, risk, low self-esteem and so forth generates an image of a diminished subject and creates low expectations of human capacities for resilience and agency (Ecclestone, 2004, 2007). Whilst a widespread emergence of what Furedi (2003) has dubbed ‘therapy culture’ implicates us all as diminished subjects, the flow of resources into interventions for emotional well-being in areas of disadvantage implies that some subjects are inherently more ‘diminished’ than others. Ecclestone and Hayes (2009) argue that interventions for emotional well-being increasingly position schools as the site for reinforcing this therapeutic ethos.

### Geography and well-being

Geographical engagements with well-being reflect a broader change in defining well-being from collective to individual (Sointu, 2005) and equating well-being with health (Atkinson & Joyce, 2011). In the 1970s, well-being research focussed on developing multi-dimensional social and objective indicators to compare territorial units (Conradson, 2012; Smith, 1973). Subsequent geographical research with objective indicators has explored inequality and social justice in how well-being relates to environmental characteristics, both built (Luechinger, 2009; Stutzer & Frey, 2008) and social (Pacione, 2003). Subjective well-being, or happiness, has emerged within geographical examinations of social inequality and the relative importance of place in determining outcomes (Ballas & Tranmer, 2012; Brereton, Clinch, & Ferreira, 2008; Morrison, 2010; Pearce, 2014). Often, research equates subjective well-being with either mental health (Fagg, Curtis, Stansfeld, & Congdon, 2006; Riva & Curtis, 2012) or priority health prevention issues such as smoking or obesity (Lake, Townshend, & Alvanides, 2010).

These geographies of well-being are implicitly based on ‘a components approach’ (Atkinson, 2013). This approach confronts and makes manageable what is inherently an ill-defined and undefinable concept by constituting well-being through sets of components, thereby tying the concept to ‘desirable’ aspects of life which can then be assessed. This approach is not limited to those undertaking quantitative investigations; qualitative research similarly positions well-being as an outcome of factors needing identification, as in the benefits from greenspace (Groenewegen, den Berg, de Vries, & Verheij, 2006) and bluespace (Völker & Kistemann, 2013). This approach treats subjective well-being as an entity that can be acquired, accumulated and retained, or indeed lost, something akin to a commodity, and associated in the wider well-being literature with the ‘telic’ approach (Diener, 2009). The approach has great practical virtue as it enables an understanding of how subjective well-being endures beyond the short-term. Moreover, allowing stability to subjective well-being makes it meaningful to measure and monitor trends and assess interventions. Change in subjective well-being can be related to change in opportunities and resources, whether external or internal to the person, facilitating the acquisition of the various components. These can be evaluated through the standard designs of before and after assessment or cross-sectional comparisons.

A second body of geographical research treats subjective well-being rather differently. This work reflects a shift in focus onto health and well-being rather than disease and medicine (Kearns & Moon, 2002; Parr, 2002), the treatment of peoples and places as mutually constitutive (Smith, 2003) and the growth of interest in emotional geographies (Anderson & Smith, 2001; Davidson, Bondi, & Smith, 2005). Research has revealed the complex relationalities of therapeutic landscapes and spaces, particularly in terms of the broader notion of subjective well-being (Conradson, 2005; Gesler, 1992; Gesler & Kearns, 2002; Tonnellier & Curtis, 2005; Williams, 2007). As the distinction between public and private spaces of care becomes blurred, research illuminates how such spaces are inseparably and mutually constituted by and constitutive of emotional experience (Brown, 2003; Davidson & Milligan, 2004; Dyck, Kontos, Angus, & McKeever, 2005; Milligan, 2003, 2005). In these strands of geographical research, subjective well-being is understood as an effect of complex relations, constituted and constitutive of both place and time, as assemblage and as always becoming. As always becoming, how subjective well-being may change is uncomplicated; instead, what is at issue is how well-being may become stable. Several theorisations offer insight as to how human existence, our bodily enactments, our relations, our ideas and, through these, our subjective well-being may become habituated; these draw variously on concepts of reflexive body techniques (Crossley, 2004), on discourse (Sparke, 2000), on governmentality and discipline (Evans, 2010), on recognition and misrecognition (Honneth, 2001) and assemblage (Deleuze & Guattari, 1987). In particular, the emphasis on assemblages of people, things, places, time and so forth allows for a culturally specified subjective well-being to be situationally and relationally both emergent and constrained (Panelli & Tipa, 2007, 2009).

### Arts and well-being

These different characterisations of subjective well-being as both stable and fluid are implicit in a model of how the creative arts may enable subjective well-being and which emphasises a complex set of interactions between arts practices and setting (Kilroy, Garner, Parkinson, Kagan, & Senior, 2007). Participation in the creative arts can enhance subjective well-being through building both an inward looking self-esteem and self-awareness and an outward looking social confidence and connectedness (Clift & Hancox, 2010; Hampshire & Matthijsse, 2010; Hillman, 2002; Kilroy et al., 2007; Parkinson, 2009) and these in turn are associated with new recognitions accorded to the participant by others across a range of different settings (Fisher, 2008; Fisher & Owen, 2008). But whilst such potential benefits to subjective well-being are documented, the ways in which the creative arts engender these effects remain under-theorised. Kilroy et al. (2007) propose that the creative arts can act as a catalyst for change by enabling the participant to become more open to new and alternative ways of being, thinking and feeling by entering a ‘flow state’. Implicit in Kilroy et al.’s model is an opposition between the habituated practices of the everyday and openness of ‘flow’. The habitual stabilises well-being but may also act as a constraint to further openness and growth, and thus, in order to enhance subjective well-being, it must first be destabilised. Such destabilisation may enable not only beneficial but also deleterious change and therefore needs careful management. The claims made for the value of participation in the creative arts is thus twofold: first, it can somehow take the participant into a space beyond the habituated routines of being and acting thereby affording openness to other possibilities; second, it can afford a space and practice in which any destabilisation of well-being is safe, being predominantly under the control of the participants themselves.

The idea of being displaced temporarily from familiar and habitual everyday routines in ways that enable new possibilities clearly resonates not only with Deleuze and Guattari’s (1987) concept of assemblage but also with their elaboration of smooth and striated space (resonant with this study they also term these sedentary and nomadic space). When these spatialised concepts are applied in an educational setting, the ritual and choreographed movements of the educational assemblage score ‘striations’ that in turn normatively define and constrain possibilities of action and meaning (Youdell & Armstrong, 2011). However, similar to Kilroy et al.’s (2007) notion of entering a ‘flow state’, assemblages can be disrupted through affective ‘lines of flight’ that escape or scatter them, enabling new becomings by allowing ‘us to trip out of the striations in which we are caught to skate on the smooth plateaus between, even if in doing so we slip into or begin to grind out yet another striation’ (Youdell & Armstrong, 2011, p. 145). Thus, the value of participation in the arts, which was apprehended through the metaphor of a catalyst by Kilroy et al. (2007), may be further developed as generating lines of flight, as tripping the participants out of the striations and into smooth, as yet uncharted, territory.

The paper thus considers how a creative arts intervention can act as the catalyst that unsettles, disrupts and destabilises well-being, and trips participants out of a performative habitus and the choreographed movements of striated space in ways that enhance rather than harm a capacity for subjective well-being. The explorations of the ways in which such tripping may occur given the specificity of assemblages draws on a case study of a school-based intervention to enhance subjective well-being through dance and movement. The paper first introduces the case study with background literature relating dance, movement and well-being and the methods for the empirical data collection. The case study section additionally describes the benefits that were perceived to have accrued to the children by those adults most closely involved in their daily lives (parents, teachers and the project dancers). The rest of the paper examines how dance and movement acted as a dynamic catalyst through three examples of moments of transition. The paper ends with concluding reflections for furthering how geographers of health approach research on subjective well-being.

## The case study

### Movement and well-being

Dance has been described as the most intimate of the expressive media in that both the feelings and thoughts expressed and the medium of expression are the total embodied self, ‘ … we make sense of our bodies first and foremost. We make sense of them in and through movement, in and through animation. Moreover, we do so without words’ (Sheets-Johnstone, 1999, p. 48). In understanding the body as a primary site of knowledge and discovery (Cancienne & Snowber, 2003; Sheets-Johstone, 1999), theorists of dance correspond with social scientists writing about movement and its relation to how our existence ‘is continually coming into being as we – through our own movement – contribute to its formation’ (Ingold, 2000, p. 242). Geographic research on mobilities, movement or dance and the connections with affect and non-representational theory recognises how movement is central to sense-, place- or self-making. Whilst such research does not explicitly address subjective well-being, it offers an expansion on a relational approach through a positively charged language of dynamism, movement, flow and continual becomings (Anderson, 2009; Duffy, 2012; McCormack, 2002; Spinney, 2006). In particular, geographical research on affect characterised ‘as a transpersonal *capacity*’ (Anderson, 2006, p. 735) or a ‘mobile energy’ (Andrews, Evans, & McAlister, 2013, p. 101) may deepen an understanding of subjective well-being as not only relationally but also affectively emergent. However, health geographers have done little with mobility apart from the instrumental benefits of movement, such as walking and exercise, for the prevention of risk factors, such as obesity (Andrews, Hall, Evans, & Colls, 2012). A similar conceptual gap is found within education regarding whether dance and movement are part of physical education or the arts (Bresler, 2004). Dance and movement clearly benefit children through physical exercise but also promote greater body awareness, focus and concentration, sensitivity to personal and social space, all of which serve to enhance subjective well-being in terms of managing emotions, self-esteem, awareness and respect for others and skills in communication, cooperation and building trust (Bergman, 1995; Deans, 2011; Hanna, 2008; Lobo & Winsler, 2006). These claims characterise the child as an embodied learner whose capacities are enhanced by encounters of and through a bodily self; the body is ‘not simply an object upon which or through which discipline or utility must be imposed, but is also that through which values, meanings and pleasures are enacted and created’ (Fensham & Gardner, 2005, p. 15).

### Methods

The case study followed an unusual creative arts project implemented in a primary school in the north of England which aimed to deliver the curriculum using dance and movement to a class of 7 to 8-year-olds (Year 3) for 3 months (September–December 2009; Yin, 2009). The research project was supported by the school and the arts-based agencies coordinating the intervention. The research was granted ethical approval through our University procedures which are compliant with ESRC and RCUK requirements and included full researcher UK CRB clearance for working with children. In keeping with standard practice to ensure informant confidentiality, all personal names have been changed and institutional identities concealed.

The case study primary school as characterised in the British government ofsted report (not referenced for confidentiality) served an urban area of significant social deprivation; as such, its involvement with the intervention reflects the attention to social and emotional well-being as part of an agenda for social justice whilst at the same time, illustrating how certain populations more than others may be characterised as vulnerable and incapacitated or diminished subjects. The school is larger than average with a pupil population of over 300. New leadership at the school had been accompanied by marked improvement in attendance and attainment. Notably, the inspection report graded as outstanding the school’s commitment to extending the curriculum to promote the pupils’ well-being through arts and sports activities. The school had other initiatives in process at the same time as the dance and movement project and had already supported other arts-based interventions. As a result, children were used to outside artists periodically coming into school to run sessions, although they had not experienced an intervention of such sustained duration.

The dance intervention was designed in three blocks, or residencies, of approximately three-and-a-half weeks, each implemented by a different dance company. Three teaching staff were directly involved in the collaboration. Harold was an experienced teacher who this year had reduced his working hours to 3 days a week. He had not taught this class before, but knew some of the children from his role in wider school activities. Helen was a newly qualified teacher who had done part of her teacher training with the same class. Mary was a Higher Level Teaching Assistant who had worked in the school for many years, lived in the area and knew the children and many of their families well. The class comprised 28 children: 18 girls and 10 boys. The school selected this class to participate in the intervention for two reasons. In England, the first term of Year 3 involves a transition from the infant stage of education to the primary stage (key stages 1–2), which it was hoped the intervention would facilitate. Second, both the school and the dancers agreed that children at this age would be willing to trying out new and alternative bodily engagements.

We followed the intervention intensively throughout its 3 months’ duration through participant observation in the classroom and school. After the first few weeks, the researcher selected eight children to accompany in greater depth. This group comprised four boys and four girls and included those struggling and those doing well at school, those engaging and those initially reluctant to engage with the dancers. The unstructured observations and informal conversations with the children were supported by more formal interviews with each of the dancers, the teaching staff and some parents. Interviews were recorded with participant permission and transcribed. The parents of 21 children were asked briefly about the project when they came into the school for a mid-term parent–teacher appointment. The researcher sent a letter to all parents in advance and spoke to the parents separately in the library. In presenting the results, reported speech is attributed by the constituency of the speaker, that is, child, parent, staff or dancer, but not given specific identifiers because the small numbers in some constituency groups would undermine confidentiality.

The field researcher typically spent 2 days in school every week during which time she participated in all activities of the classroom and made unstructured observations and personal reflections:

When the children came in, Harold introduced me as ‘This is [name], who is interested in what we are doing and will be spending some time with us.’ The children then all said ‘Good morning [name]’. There was no real opportunity to introduce myself properly and to explain who I was, where I was from and why I was there. It bothered me a little but none of the children questioned who I was then or afterwards. They seemed to accept my presence, some wanted to sit next to me. I thought that in time I would tell the children what I was doing. Initially I did everything the children did and joined in with the activities, sometimes helping them, sometimes participating myself. I tried to blur roles so that I wasn’t perceived as a dancer or as a teacher, just a friendly adult. [from Field-notes]

The children quickly became accustomed to the presence of the researcher; she sat with them on the floor cushions and joined in the activities:

Initially I had an A4 book but the children were given an A5 book by the second residency as a reflection book and so I bought a similar one and when the children are asked to reflect in their books I get mine out and write some notes. I have also made a point of leaving my book open and in view when writing notes, leaving it in the classroom when I am not there and showing both children, practitioners and teachers things I have noticed by letting them read it in my book. I am trying to instil a joint ownership of these observations, being open and transparent. I have told everyone quite clearly they can look in the book at any time. I do not write anything in it that I wouldn’t be happy anyone reading. I feel this initial work on building trust has now paid massive dividends as I can now write notes without me or others feeling uncomfortable as the notebook is seen as an open record. The children like it when I write down what they dictate in my book and they sometimes want to see how many pages they have filled and when I show them it always looks like a lot to them and they always seem pleased. [from Field-notes]

We had a particular interest in the relational, spatial and affective dynamics of the class, but, similarly and extending the experience of other researchers (see Andrews et al., 2013), we found capturing either ‘affective happenings’ or emotional expression not only methodologically challenging but also ethically complex. In making observations, we discussed extensively the risks of drifting into a kind of ‘pop-psychology’ if we began to ascribe and interpret an affective happening or emotional reaction to the movement or expressions of bodies beyond simple categories of smiling or upset. In reality, affects or emotional expressions have to be interpreted through a cognitive filter, whether the child’s own reflections, or interpretation by the researcher or another adult informant (McCormack, 2003; Pile, 2010). We tended to privilege the insights and observations made by the project dancers given their particular expertise and attunement to the movement and expressiveness of bodies. Nonetheless, we were comfortable ascribing affectivities within a series of tensions between the practices of the dancers and those of the teachers which were evident not only from observations in the classes but also from the reflections of dancers, staff and children. We therefore draw on the observations, conversations and interviews to provide an account of the emergence and significance of some of these tensions in relation to enhancing the children’s subjective well-being.

### Enhancing subjective well-being

The school sought the intervention to both improve subjective well-being and contribute to pedagogical objectives through increased motivation in learning and experimentation with non-sedentary approaches to learning. Critics, such as Ecclestone and Hayes (2009), have argued that the inclusion of attention to well-being within the school curriculum diminishes both the human subject and the subject content. This study did not provide much support for this argument. Certainly, as regards the subject content, the teachers asserted their focus on learning outcomes rather than a well-being agenda and expressed repeated anxiety about the loss of pace and breadth in learning. Nonetheless, despite their evident reservations and prioritising of learning outcomes, the teachers did consider the project highly successful in terms of raising children’s subjective well-being:

I did notice how much they trusted each other … It was good to see them do it. [Teacher]

I’ve watched her week by week grow in confidence. [Teacher]

Hannah … has lots of problems but … she has succeeded in so many different ways and I think she has felt that success. [Teacher]

Perhaps more interestingly, despite reservations about the benefits to learning, the teachers’ recognition of gains for subjective well-being included the indirect factors commonly assumed by teachers to be necessary for a successful learning environment such as focus, concentration, enthusiasm, happiness, having fun and being fully engaged. There was then, perhaps, an artificial separation in the teachers’ approach between those activities supporting the curriculum and the learning outcomes and those supporting subjective well-being.

Parents also indicated that subjective well-being had improved in several cases. Children who had been reluctant and unhappy to go to school were now eager to go:

he used to hate coming to school last year, used to have to drag him kicking and screaming, there is no problem coming to school now, he gets up gets dressed. [Parent]

Many parents reported their children’s increased enthusiasm and energy, not just for school, but for doing more in general:

She’s doing more at home because she’s repeating stuff she’s done at school, comes out with new things all the time, more than last year, she’s got more energy now, she’s always upstairs making stuff and that’s different to last year. [Parent]

Moreover, almost half had noticed increased activity levels or changes in the nature of the activities their child engaged with:

Now she is constantly on the go, it’s like she’s got a zest for playing out. Before she would only play out for half an hour, now she’s out all the time, can’t get her in. [Parent]

and a few parents noted improvements in problematic behaviour patterns:

We used to always get called in about him hitting people and bad attitude but that’s completely stopped, well there’s little bits of trouble but not to the same extent. [Parent]

Half the parents considered that their child had gained confidence, particularly in their social interactions. And the most striking observation made by parents was how much more the children were now talking to them and to other adults:

He’s different this term, he’s talking more; he is just more open and outgoing, normally he’d just be quiet after school. [Parent]

I’ve noticed she’s using bigger words and her vocabulary has increased, she talks more about what she’s done, she talks about the project at home. [Parent]

A range of benefits in children’s subjective well-being is thus evident from the project. The next section offers a description of the work of the different dance companies in the school before exploring the ways in which such work could act as a dynamic catalyst for well-being.

## Intervention through dance and movement

Three dancer residencies took place through which artists worked with the class teachers focussing on ‘non-sedentary’ approaches to teaching, learning and enhancing subjective well-being. Each of the three residencies approached the brief with different philosophies and established different working relationships with the teaching staff and children.

The first residency had a focus on dance, play and creative movement with young children, talking of movement rather than dance:

we were using the word movement, it’s about embodied learning, understanding things through your body and using movement to communicate and explore a particular topic.

The company had a strong performance element to their work, particularly in aerial dance. Their resources included trapeze and suspended fabric pouches which people can climb inside called cocoons. They aimed to encourage creativity, curiosity and confidence by using the excitement of movement to engage the children and to provoke a gentle playfulness centred on body awareness:

… in terms of education work I think children know lots about letting their creative ideas flow, letting their ideas be embodied and visible and a lot of what we are doing is creating times and spaces to really value what they are doing.

Their approach aimed to allow children space and time to ‘be in their bodies’ to feel and notice. This residency looked for ways to be more aware of movement in everyday routines of the school as well as creating movement responses to curriculum concepts. For example, they used registration as a way to gradually encourage movement and confidence, so children would call out their names in a circle and perform an action. Whilst registration took longer as the teachers had to find names on the register rather than just read them in order, it was noticeable that after a few sessions, the children became less reticent and their movements became more confident and expressive.

As the first residency, the dancers had to negotiate the wariness and anxiety of the teachers to build a working relationship and as such effectively sounded out the possibilities, involved other staff and parents and successfully initiated trust in the potential of the intervention. Moreover, the dancers were concerned not to undermine the teachers whilst the teachers acknowledged that they reverted to conventional teaching modes when the dancers were not in (Friday). This cautious approach meant that their inputs were largely reactive to and incorporated into the school curriculum; the teachers would deliver a lesson and invite the dancers to do some movement-based activity to reinforce it. Some parts of the curriculum lent themselves very well to dance movements such as exploring symmetry, shadows and literacy.

The second residency was far more assertive in taking over the classroom and involving the teachers as participants and assistants rather than responding to a teacher-led agenda. They encouraged pupils to take responsibility for their own learning and to spend time listening, responding and documenting the process with the children using diaries and film. They preferred to work with the term non-sedentary rather than dance:

We were drawn to the word ‘non-sedentary’ because the way that we work across art forms means we don’t compartmentalise … that is how we approach movement.

Their broad interpretation of what the project was about enabled them to be much freer in terms of how they responded creatively to the challenges of the curriculum. Although they had a drama/dance trained artist and a visual artist, they worked across a range of art forms including not only movement but also music, sculpture, painting, textile and outdoor art and drew on a wide array of material resources, including natural materials, theatrical and historical costume and artefacts, and kites.

The teachers viewed this residency as the most successful for several reasons. First, roles were very clear; the dancers took the lead giving the teachers both experience in supporting and the opportunity to observe. Second, planning was meticulous in ways the teachers recognised, so they felt reassured that learning objectives were covered and were confident handing over preparation for lessons to the dancers. Third, the company’s broad interpretation of movement provided a wide range of opportunities for the children which teachers could more easily link to their learning objectives. Fourth, they introduced diaries for the children and encouraged the children to document what they had learnt which eased teachers’ anxieties about recording. Fifth, because the dancers always worked together, they were only present in school for 3 days. This meant that on the other 2 days, teachers were able to deliver conventional lessons and do all the things that they felt necessary to keep the children ‘on track’. Lastly, the quality and distinctiveness of the company’s work captured the teachers’ imaginations and surprised them in terms of what the children were able to achieve:

… the planning was up front, I always had it the week before or the weekend before, I knew exactly what they were doing in terms of it being written on paper, they made cross curricular links which is fantastic. Immediately I knew my role and it was as a supportive role … I think what they did was amazing in many ways, some of the experiences that the children had were first class really, it was obvious that they put so much work into what they did, I just think the children had a really nice experience with them. I sat back at times and just watched what was going on and it was absolutely fabulous … [Class teacher]

The cross-curriculum approach was exemplified in a kite making project. The dancers brought in a large kite and introduced the project by working with the children in the hall to interpret the movement of the kite and express it artistically through writing, dance and mark-making. They talked with the children about scientific principles of gravity, lift and wind direction. Back in the classroom, they made a kite from scratch over 1 day. They folded a square of paper and dipped it into inks, discussed properties, such as absorbency of the materials and colour combinations. The pattern revealed when the paper was unfolded related to the children’s learning of symmetry. The children also examined how to make the frame of the kite and tie the slip knots as a contribution to design and technology. Finally, they had a celebration where, in perfect kite flying weather, they all flew their 27 colourful kites on the school field, brilliant against a clear blue sky. One child said that it had been the best day of her life.

The third residency was much more exploratory and experimental and less obviously well integrated into the core curriculum. Moreover, given the previous residency had built a strong, positive relationship with the teachers and resonated with their need for detailed planning, this more exploratory approach was quickly judged unfavourably in comparison. In addition, the third residency encountered a marked slump in energy for the project as a whole. The teachers reported that they were starting to get tired of the lack of structure and wanted things back to normal. Similarly, many of the children were physically tired and also wanted their desks back. The dancer introduced a green screen and film, with the children crawling across it and then drawing in their own environments which he was able to link to the curriculum on Victorian life. The residency also contributed directly to the well-being agenda in terms of building confidence and engagement, particularly through a project on bullying.

A characteristic of his residency was working with quite high energy movement, allowing the children to be excited, loud and expressive which contrasted markedly with the much quieter and gentler approach of the previous residency and was perceived negatively in terms of class control by the teachers:

This residency has been the least effective, there was a big drop in the quality of their (the children’s) involvement, I could see the behaviour of the children going downhill.

He put more emphasis on solo performance with each child being expected to ‘have a go’ in front of the rest of the class whilst other children waited their turn. The greater emphasis on teaching dance performatively, getting the children to work on choreographed routines and working their bodies in particular ways, was exciting for some and especially those who had been disappointed earlier because they had thought the project was going to teach them dance. Thus, some children found the opportunity to perform in this way to be genuinely exciting and resonated strongly with their natural preferences. Others found that being pushed showed them what they could achieve which they clearly enjoyed despite initial shyness and embarrassment. But some children did appear to feel exposed and found ways to opt out. The dancer also provided an acceptable and ‘cool’ male dancer role model for many of the boys in the school. Whenever he walked into the schoolyard, he was often surrounded by children, particularly boys, asking him to show them street dance moves and, as such, he succeeded in interacting with children across the whole school far more than the other residencies.

We have described the residencies in some detail as it is in the tension between the habituated routines of classroom and the alternative approaches of the dance residencies that different modes of disruption, tripping and flight may emerge. The sequence of the three residencies established a progression from movements supplementing a teacher-led agenda in a somewhat tentative manner, to movements leading the learning process but through highly structured planning, through to movement and dance as experimental and performative and as diverging from pedagogical goals apart from the social and emotional agenda.

### In the classroom

Geographers have examined the ways in which buildings, architecture and the other material objects associated with them may choreograph the everyday practices and affective states of those who thereby inhabit and in turn constitute the space (Kraftl, 2010; Lees, 2001; Merriman, 2011). Dancers have likewise noted how spatial arrangements reflect and facilitate desired relational dynamics, whether between teacher and pupils or between pupils when working together (Bresler, 2004). The space of the conventional classroom constitutes an assemblage that is striated with multiple lines of material order in equipment and furniture, of disciplined conduct of movement and sound in sitting, hand-raising and listening, of managed time and duration into schedules of order and play, of direction and goals in curricula and targets for the successful child and of teacher control through expertise and authority. The project disrupted the assemblage of the everyday primary school classroom both literally in re-assembling the material components, relationally in enabling different movement and interactions and affectively through shifts of discomforts and excitements. The evident tensions arising from this unsettlement may also be apprehended as affectivities, as flows or lines of flight that escape and exceed the constraints on subjectivities.

The removal of the tables and chairs from the classroom and cushions and the installation of a dance floor provoked considerable controversy. The absence of tables meant that children had much greater freedom to position themselves where they wanted in the physical space of the classroom. This made for an element of disorder and the dancers recognised that for the teachers, the structure of the traditional classroom environment was closely linked to the idea of structured learning:

I think the fact that there were no tables and chairs changed the face of it, it felt like it didn’t have as much structure. [Dancer]

The tables and chairs had provided a structure to facilitate organisation and control. Now they were unable to group children in terms of their different learning needs and they were unable to prepare class materials and place books on tables in advance during breaks. This meant considerable extra time giving out books eating into already constrained lesson time. But even as the teachers reacted, the affective tension ‘tripped’ them into new terrain as they began to question basic elements in their practice:

There was also an issue with fiddling with the cushions and this started a discussion about the perceived link teachers have between fiddling and not paying attention. Helen said it is drummed into you at teacher training to get the class to stop fidgeting and look at you but this doesn’t necessarily mean they are paying attention. But she said she knew that but still yelled at the class to stop fidgeting. ‘Have got to get out of the mindset of getting them to sit still and listen, examiners on your teaching practice will tell you to get the kids to keep still.’ Helen feels she tells the children to ‘shhh’ all the time ‘I’m a complete control freak!’. [Field-notes]

Teachers also experienced the benefits of being able to move around the class more easily and the different classroom dynamic of getting down to the children’s level. They enjoyed the greater ability to observe children and talk with them during the project. The dancers experienced no similar tension; they all felt that clearing the room allowed for more intense, focussed work and an opportunity to develop physical, body, movement relationships and learning:

Not having furniture in the classroom gave us a lot of time on the floor next to the children in physical moving contact, that was just interesting in terms of them feeling comfortable with me, with their bodies, seeing someone else comfortable in their body.’ [Dancer]

The children also had mixed responses to the disruptions to their classroom space. They mostly seemed to enjoy the space and the cushions and made themselves comfortable in various ways. They too may be ‘tripped’ out of the striated space of the conventional classroom, and, whilst happy during the new activities, expressed their greatest discomfort when engaged in more conventional school work. As Deleuze and Guattari note, ‘Of course, smooth spaces are not in themselves liberatory’ (1987, p. 581) and such unsettling of the habitual is not automatically positive; one child reacted very badly at first to the reconfigured classroom:

she couldn’t get her head round the way things were working … to her that wasn’t an everyday school day, this wasn’t school life, going in without tables and chairs. [Teacher]

At the same time, those who do well in the school environment also wanted what was familiar – an affective flow that in some cases literally moved them:

After break the children worked with Helen in the classroom doing English and writing their postcards. It was interesting as the children were writing their postcards on the floor or on clipboards and Helen asked if anyone wanted to sit at a desk to write as there are a couple of desks in the corridor. Many hands shot up, mainly from the brighter girls. So four girls were chosen to sit outside. I talked to them and asked them why and they said it was uncomfortable to sit on the floor to do writing and it hurt their backs. [Field-notes]

These affectivities may be seen both as resistance to disruption and reassertion of presence within familiar striations or as lines of flight from an emerging new striation.

### Spectacle, curiosity and performance

The desk-free space of the classroom allowed movement and actions for learning and skill development in vastly different ways to the conventional pedagogic modes. Three examples, one from each residency, illustrate the ways in which the differently configured materiality of the classroom and the different practices of interaction between the children and the dancers are accompanied by affective flows that disrupt assemblage. Such disruptions occur in ways that may enable participants to flee its striated familiarity into a discomforting smoothness, to initiate new striations or, perhaps less radically, to rework existing grooves.

She talked to the group about how you don’t just listen with your ears but your listen with your whole body. Then she did a dance where she moved around within the circle and came very close to the children and looked at them and then moved away again turning and rolling on the floor in a very fluid way. The children really enjoyed this, the interaction made them giggle and they seemed very enlivened by watching her movements. Then she stood in the middle of the circle and asked for a volunteer to help her show how you listen with your whole body. She chose a boy and asked him to watch carefully and mirror her movements and he did. He concentrated very hard and the class was very quiet watching them. Then she asked him to take over and she would copy him, he struggled more with this and the movements became more constrained. Then she withdrew and asked for another child to partner the boy first one copying the other and then at some point changing over. Sometimes the children weren’t sure who was copying who and they had to really visually communicate with each other. [Field-notes]

The mirror exercise provokes the children towards sensitivity to, and a close concentration on, the spatial relation of one’s own body to the movement of another body. But the majority of the class are in fact spectators in this exercise and are not themselves moving. And yet, they appear mesmerised, entertained and ‘enlivened’ through the act of watching and listening. They are accustomed to sitting quietly to watch and listen, at least for short spells, in the conventional classroom, but here the assemblage of the classroom, the ‘teacher’, the actions and the watching and listening is unfamiliar, demanding or permitting new conduct. The person in the teacher’s place in the assemblage moves dramatically differently, constantly remaking the relations of her body to the children and the circle. And whilst the children attend as would be expected in the conventional teacher–pupil setting, the uncommon movement of an adult body moves affectivities that escape a conventional pupil subjectivation through giggles, voluntary silences of concentration and new enlivenments.

Then, again in silence, Lesley took out the basket of pebbles again, put them in front of the child next to her and motioned to the two pebbles. The child knew straight away she had to take two pebbles and put them down in front of her and pass the basket on. There was some gentle music playing while all children took two pebbles out of the basket and put them in front of them on the floor. I was surprised that no child touched or fiddled with the pebbles at all while they were waiting. There was an air of expectation. I found the exercise of taking the pebbles out of the basket in turns (I joined in with this) strangely gripping. Then Lesley stood up slowly, picked up her pebbles and placed them in the centre of the circle and sat down. She looked at the child next to her and motioned she was to do the same. So the children started to put their stones in the middle in turn. Two parallel lines of stones started to form. When Georgia’s turn came she placed her pebbles to break up this pattern, then the children after her put their stones in a more random position. Then Lesley put a strip of thin white tape over the stones, it had the word ‘symmetry’ written on it over and over down its length. They asked the children to go around again and move two stones (any stones) to make a symmetrical pattern using the line of symmetry. Some children obviously understood clearly and others seemed very unsure but everyone attempted to move two stones. Some gentle music played in the background and the atmosphere was very meditative and curious as the stone pattern was changing and emerging. [Field-notes]

Again the dancers provide a spectacle for the children initially but which, gradually, the children are invited to join. The desk-free space allows both movement of people and the creation of a floor pattern with the pebbles. This residency liked to work without or beyond words, radically different from the conventional classroom engagement, and thus explicitly draws out movement, observation and interaction as modes through which children discover for themselves. Here, as in the mirror exercise, the children are enthralled and stilled, surprisingly not fiddling with the pebbles in the more casual configuration of the room. Explicit attention has been paid to managing the flow of affectivity of the space; the potential discomfort of silence is managed through the use of gentle background music, a literal and affective flow of atmosphere. The children feel the pebbles in their hands and move to contribute to how patterns are changing and emerging from their own actions, actions which in turn attract their curiosity and attention in a way that becomes meditative. It is the movement of pebbles and children that is foremost, rather than the lines of the patterns that emerge, resonant with smooth space in which points and lines are subjugated by the trajectory, filled with events rather than formed or perceived things (Deleuze & Guattari, 1987). And yet, whilst the children are free to place their pebbles where they want, they imitate the first movements by choice illustrating how easily practices become habitual. Then again, as a pattern starts to emerge, a child intentionally disrupts this becoming of the habitual and initiates a different mode of placing the pebbles which again others imitate. Gently, the children are eased from their habitual striated space but, through imitation, effectively gently fall into a new, perhaps more lightly striated space enabling learning through these different encounters with things, shapes and numbers. In this case, we can apprehend complexity in the relations of the habitual and openness; whilst the exercise as a whole disrupts assemblages of the material, affective and relational, eliciting something of a meditative flow experience, at the same time smaller movements between imitation and innovation, striation and smoothness, repeatedly occur.

The dancer asked the children to get into pairs and taught them how to do counter-balancing. Each pair stood side by side and held hands and leaned away from each other. The children practised this for a while and then showed the rest of the group in turns. This counter-balancing formed the basis for choreographed piece around bullying and bystanders which the two dancers worked on with the children for a performance to the whole school. The piece centred on the idea that sometimes it’s difficult to help someone in trouble because you are scared. The piece represented a victim being pushed into the ground by a gang of bullies by them all putting a hand on the top of her head and gently pressing her down, powerfully conveying the mental as well as physical impact. The ‘victim’ held out her arm to people walking by, some of whom held her hand and leaned back to pull her up, using the counter-balancing skills they had learnt, but let go at the last minute through fear and so she fell back down again. The children worked very hard and applied themselves to learn the particular quality of movement required and seemed to get satisfaction out of this.

The final residency gave explicit attention to movement as performance. Here, the children themselves provide the spectacle, first for themselves and one other while experimenting with counter-balancing, second for the whole class in demonstrating their counter-balancing and lastly for the whole school in a choreographed piece on bullying. The physical exercise of counter-balance directly enables both trust and strength between the children which is then transposed symbolically through the performance. Here the flow of affectivity is explicit in the attention given to the relationality between children and the complex assemblages that may shape action or inaction. And again, the children are fully engaged, applying themselves, enjoying themselves and with direct relevance for subjective well-being, feeling good about the performance. The content of the exercise speaks directly to an established agenda for subjective well-being in schools and as such the choreographed performance carries relevance across the school. A drama performance is also recognisable as a conventional school-based activity compared with the use of movement in numeracy or design and technology. Here, the dancers are arguably less directive than in the second and even the first example; although they have defined the topic and initiate the explorations of trust and strength through the counter-balancing exercise, they facilitate rather than direct the emergent movements that enable performers to feel or inhabit the trusting, strong or bullied body. In this case, the benefits to subjective well-being may come mostly from the satisfaction of performance and the recognitions of others – a shared, decentred and collective well-being that endures not so much through the complex emergence of a new assemblage but rather through re-workings of small recognitions within an existing status quo.

## Destabilising and restabilising well-being through the arts

Deleuze and Guattari offer insights for understanding the processes through which arts and health projects bring benefits (see Fox, 2013), especially for our central concern with destabilising and restabilising subjective well-being.

The case study of a dance and movement project disrupts, unsettles or deterritorialises the striations of ‘proper’ conduct of bodies, spaces and affects within the primary school. The specific material and affective relations of a primary classroom are built on a well-established repertoire of habitual practices, including highly controlled movements in order to enable children’s learning. The altered material arrangement of the classroom directly insists on new engagements with the space, the objects of the room including the floor and between the people in that space. The dancers enable, rather than constrain, movement in that the children are expected to learn through the movement of their bodies, not despite it. The dancers bring new objects with which to interact, use much more visual techniques, introduce music and rhythm into learning and thereby de-emphasise the conventional privileging of spoken and written language. The potential of the intervention for ‘tripping’ children out of the habitual and into a more open space of possibility through moments of material, experiential, affective encounter that scatter or escape habituated assemblages seems limitless.

At the same time, as children and teachers confront the uncertainties of conduct within the new emerging assemblages of the classroom, new striations are immediately established, sometimes restoring previous striations (the dancer replacing the teacher as the one who leads), sometimes patterning new (uncertainty calmed through imitation). As such, the opposition between striated and smooth space is reaffirmed as a useful analytical abstraction but one not to be overworked: ‘ … the two spaces in fact exist only in mixture: smooth space is constantly being translated, transversed into a striated space; striated space is constantly being reversed, returned to a smooth space’ (Deleuze & Guattari, 1987, p. 552).

The value of this abstraction in relation to intentional intervention is the opportunity to apprehend the practices through which movements between the two spaces afford beneficial transitions between destabilised and restabilising subjective well-being: ‘You may make a rupture, draw a line of flight, yet there is still a danger that you will reencounter organizations that restratify everything, formations that restore power to a signifier, attributions that reconstitute a subject’ (Deleuze & Guattari, 1987, p. 9).

The concept of assemblage and the striation of space enable an understanding of the status quo, how practices and relations become routine and habituated. But they also afford, through the notion of smooth space or surfaces, a way of imagining an unmarked terrain that resonates with and perhaps offers a more useful term to Kilroy et al.’s (2007) notion of flow. The attention to affectivity and lines of flight apprehends of temporary escape from the habitual into a smooth space of possibility, transition and the emergence of new assemblage. Our first example evidences how the habituated expectations of classroom conduct are disrupted through the different engagements of the intervention, generating an affective tension expressed through giggles, concentration and enlivenments. The second example, however, is more complex with multiple moments combining what might be seen as movements in and between striated and smooth space. And the third example would appear to draw its benefits expressly from action within an established mode of dramatic performance but within which possibilities for moving into smooth space of openness is enabled through the recognitions of others. As such, the metaphor of striated and smooth spaces continues to offer insight in imagining both the constraints on subjective well-being and the possibilities afforded by participation in the creative arts. However, the complexities of the simultaneous interplay of these different forms of space serve to diminish the significance of imagining a critical moment of transition, of tripping, of escape or of scatter. Instead, we suggest that greater attention is required on the interlacing of habit and openness, and on how these may enable one another. For some children, the acts of imitation that contributed to gently striating the unknown, unmarked and possibly uncomfortable, smooth space of a new classroom assemblage may have been exactly the acts that enabled new possibilities for their well-being.

A second observation, then, on uses of Deleuze and Guattari’s work is to challenge a tendency to emphasise the dramatic moment, the explicitly intense affectivities of tension, conflict, struggle as in the two educational examples given by Youdell and Armstrong (2011). The first two events described in our study convey an affective atmosphere of gentleness, transitions between striated and smooth attended by affectivities of meditation, concentration and enchantment; the third was more explicitly energetic and in tension with the striated space of the school. This observation insists on greater attention to the gendered nature of transition or lines of flight in Deleuze and Guattari’s analyses. Again, both examples in Youdell and Armstrong involve male protagonists; in our study, the first two residencies were females, the third male. Feminist analyses on other topics have challenged the universal experience of sudden moments of transition as masculinist and gendered (see e.g., Walker Bynum’s work on liminality, 1991). Deleuze and Guattari’s work has informed studies exploring the gendered nature of affective assemblages and subjectivities (e.g.,Ringrose, 2011) but the affectivities of transition, the lines of flight, and of deterritorialisation demand greater critical attention to the necessity of the sudden momentariness of trip, of scatter and of rupture (see Tamboukou, 2008, who also nods towards this).

For geographers of health, the interest is less on the production of space as our primary focus, but more on foregrounding the normative and political questions related to how we enhance or damage human well-being. Our account of how a dance and movement intervention may have generated observable gains in subjective well-being has clear implications for how well-being is understood and how interventions are prioritised. Here, the assemblage is where the intervention has its impact, intentionally unsettling habituated practices and relations but not in prescribed or predictable ways. The increasingly widespread use of interventions for subjective well-being designed to build the internal resources of individuals in terms of resilience, positivity and fulfilment, largely miss the importance of ‘the ways that subjects are constituted as well as the ways that these are unsettled both through the practices of subjectivated subjects and through the flows of affectivities that exceed these subjects and the striations of space’ (Youdell & Armstrong, 2011, p. 150).
